# Predictors of Green Behavior Among Hospital Nurses: Organizational Climate, Team Climate, and Awareness of Consequences

**DOI:** 10.1111/inr.70203

**Published:** 2026-06-22

**Authors:** Seung Eun Lee, Heejin Lim, Sujin Nam, V. Susan Dahinten

**Affiliations:** ^1^ College of Nursing Mo‐Im KIM Nursing Research Institute Yonsei University Seoul South Korea; ^2^ College of Nursing Yonsei University Seoul South Korea; ^3^ School of Nursing University of British Columbia Vancouver Canada; ^4^ Faculty of Nursing University of Tabuk Tabuk Saudi Arabia

**Keywords:** employee green behavior, environmental awareness, environmental sustainability, healthcare, nurses, prosocial behavior, working conditions

## Abstract

**Aim:**

This study aimed to examine the relationship between nurses’ perceptions of the green organizational climate, perceptions of green team climate, and awareness of consequences, are related to their green behavior in hospitals.

**Background:**

Healthcare is a resource‐intensive sector where everyday employee green behavior can meaningfully reduce environmental impacts. Nurses, who work at points of concentrated resource use and waste generation, are pivotal for sustainability in hospitals. Yet, factors associated with nurses’ green behavior remain underexplored.

**Methods:**

This study adopted a correlational, cross‐sectional design and is reported in accordance with the STROBE guidelines. Data were collected from 649 nurses working in six hospitals in South Korea through an online survey conducted in January and February 2025. Multiple linear regression estimated associations between the three predictors and green behavior, controlling for age, conscientiousness, and direct care provision of nurses.

**Results:**

Green team climate emerged as the strongest predictor of nurses’ green behavior, followed by awareness of consequences and green organizational climate.

**Discussion:**

Team‐level environmental norms may have a stronger influence on nurses’ green behavior than broader organizational climate. Awareness of environmental consequences also contributes to green behavior.

**Conclusions:**

Nurses’ green behavior was positively associated with both contextual climates and individual cognitions.

**Implications for Nursing:**

Promoting pro‐environmental team norms, enhancing consequence awareness, and aligning organizational practices with sustainability priorities may encourage green behavior. Peer‐led environmental initiatives at the unit‐level and consequence‐awareness training can strengthen shared norms among the team and promote green behavior.

**Implication for Health Policy:**

Hospitals should embed environmental sustainability in policies, performance management, and operating procedures, including procurement and waste management. At the larger systems level, environmental metrics could be tied to accreditation and funding.

## 1 Introduction

As environmental sustainability emerges as a global priority, the healthcare industry, which is characterized by high resource consumption and significant waste generation, faces increasing pressure to adopt environmentally responsible practices (Sahoo et al. 2025). Within this context, employee green behavior plays an important role in supporting organizational environmental goals in the workplace (Ones and Dilchert 2012). In hospital settings, green behavior includes everyday practices such as proper waste segregation (Dolcini et al. 2025) and minimizing the use of environmentally harmful materials or procedures (Olawade et al. 2025).

Among healthcare professionals, nurses are well‐positioned to advance sustainability because they work at many points where resources are used and waste is generated, and they exercise significant day‐to‐day discretion over consumption, reuse, and waste segregation (Wohlford et al. 2020). As visible and trusted team members, nurses can also model and diffuse pro‐environmental norms (Luque‐Alcaraz et al. 2024). Consequently, their green behavior may meaningfully affect hospitals’ environmental performance. However, the factors associated with nurses’ engagement in green behavior remain underexplored, particularly regarding how organizational, team‐related, and individual factors may operate concurrently to influence employee behavior, leaving an important gap in the literature on sustainability in healthcare (Chung et al. 2024). Although healthcare systems and environmental policies vary across countries, environmental sustainability is a global problem, and promoting environmentally responsible practices among healthcare professionals is likely to require attention at multiple levels across hospital settings. Thus, in this study, we aimed to identify key factors that influence green behavior among nurses, offering insights that may inform more effective sustainability initiatives in healthcare organizations.

## 2 Background

A growing literature identifies green behavior as a lever for organizational sustainability (Zacher et al. 2023). Yet, most studies have examined general organizational contexts, and the healthcare sector remains underexplored. While sustainability efforts in healthcare have prioritized technological and systems‐level solutions (Olawade et al. 2025), there is limited evidence on the factors that influence nurses’ green behavior. Building on a multisector review showing that green behavior relates to both contextual and individual factors (Zacher et al. 2023), this study focused on three potential correlates of nurses’ green behavior in hospital settings: green organizational climate, green team climate, and awareness of environmental consequences.

Green organizational climate refers to employees’ shared perceptions that their organization values and supports environmental sustainability (Norton et al. 2014). The organization's pro‐environmental goals may be conveyed through formal policies, operating procedures, and management practices oriented toward sustainability (Norton et al. 2012). Prior studies have shown that when sustainability is emphasized by the organization, employees perceive green behavior as socially desirable, leading them to align their behaviors with these expectations (Aly et al. 2025; Thabet et al. 2023). Guided by this literature, we examined whether nurses’ perceptions of a green organizational climate were positively associated with their green behavior.

The immediate social environment within work teams is also an important factor in understanding employees’ behavior. Green team climate denotes employees’ perceptions that co‐workers endorse and enact environmentally responsible practices (Norton et al. 2014). In contrast to organizational climate, team climate reflects more proximal and informal influences that are communicated through everyday interactions and shared norms among co‐workers within teams. When environmental sustainability is perceived as a team norm, employees tend to conform to those norms and model their peers’ behaviors, thereby increasing pro‐environmental behavior in the workplace (Norton et al. 2014, 2015). A recent review by Zacher et al. (2023) stated that team climate is an antecedent of green behavior across various occupations, but evidence from hospital settings remains limited. Given the collaborative, interdependent nature of nursing work (Rosen et al. 2018), where peer norms guide daily routines, we hypothesized that green team climate would be positively associated with nurses’ green behavior.

While organizational context and team‐level social norms are associated with green behavior, cognitive factors such as environmental awareness are also important. According to the Norm Activation Model, individuals are more likely to engage in prosocial or environmentally responsible behavior when they are aware that their actions may have harmful consequences for others or the environment (Han 2014; Schwartz 1977). This idea is captured by the concept of awareness of consequences, defined as the extent to which individuals recognize the potential harm of their behaviors (Dalvi‐Esfahani et al. 2017). In line with the Norm Activation Model, we conceptualized awareness of consequences as a cognitive precursor that heightens sensitivity to the environmental impact of one's actions and facilitates the activation of moral norms. For example, individuals who believe that their actions contribute to global warming or climate change may feel a stronger sense of moral responsibility, which can motivate environmentally friendly behavior (Onwezen et al. 2013). Fu et al. (2020) and Li et al. (2023) found that awareness of consequences was positively associated with employee green behavior; however, this relationship has received limited attention in healthcare settings. Accordingly, we investigated whether nurses’ awareness of consequences was positively associated with their engagement in green behavior.

**FIGURE 1:**
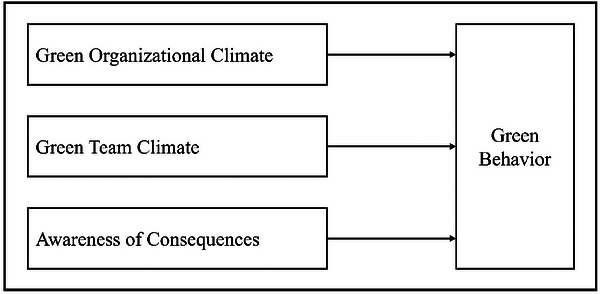
Conceptual framework. The framework illustrates the hypothesized associations between nurses’ perceptions of green organizational climate, green team climate, awareness of consequences, and their engagement in green behavior.

This study examined three theoretically grounded constructs, green organizational climate, green team climate, and awareness of consequences, to assess their relevance to nurses’ engagement in green behavior (see Figure 1). By doing so, in this study, we aimed to extend existing sustainability research into the healthcare domain by offering an integrative examination of organizational, team‐related, and individual correlates of nurses’ green behavior.

## 3 Methods

### 3.1 Study Design, Setting, and Participants

Data were obtained through a cross‐sectional online survey conducted in January and February 2025 across six hospitals in South Korea. These hospitals were selected based on accessibility and willingness to participate, and all participating hospitals provided acute inpatient care, offering a common clinical context for nursing practice. Convenience sampling was used to recruit eligible participants, defined as nurses with at least six months of clinical experience, with no additional exclusion criteria. At each participating hospital, the survey link was distributed by nursing department staff responsible for research coordination and shared with all eligible nursing staff. Because the survey link could be forwarded within hospitals, the delivery rate could not be determined, and a response rate could not be calculated. Of the 942 individuals who accessed the survey, 650 completed it. One response was excluded because of a straight‐lining response pattern (i.e., selecting the same response for all items), resulting in a final analytic sample of 649 nurses. A power analysis using G*Power indicated that the final sample size was sufficient to ensure a statistical power of over 0.9 for multiple regression analysis with six predictors, based on a type 1 error of 0.05 and a small effect size (Faul et al. 2009).

### 3.2 Study Variables

This study examined green behavior as the dependent variable, with green organizational climate, green team climate, and awareness of consequences as independent variables. Responses were rated on a 5‐point Likert scale (1 = *strongly disagree*, 5 = *strongly agree*), with higher scores indicating greater levels of the construct.

#### 3.2.1 Green Behavior

Green behavior was measured using a five‐item scale originally developed in Korean by Chang et al. (2023). A sample item is, “In the workplace, I sort recyclable materials.” The original scale demonstrated good reliability and construct validity and showed a Cronbach's alpha of 0.74 (Chang et al. 2023). In the present sample, the alpha value was 0.62, which exceeds the minimum threshold proposed for acceptable internal consistency in applied research (Ursachi et al. 2015).

#### 3.2.2 Green Organizational Climate

Green organizational climate was assessed using a three‐item scale originally developed in Korean by Chang et al. (2023). To suit the hospital context, minor wording changes were made, such as replacing “company” with “hospital,” to adapt the original items, which were designed for corporate settings. To examine whether the adapted items retained an appropriate measurement structure, we conducted exploratory factor analysis (EFA), which supported a one‐factor model. An example item is, “Our hospital is interested in supporting environmental causes.” The original scale demonstrated a Cronbach's alpha of 0.91 (Chang et al. 2023); the reliability coefficient was 0.93 in the present sample.

#### 3.2.3 Green Team Climate

Green team climate was assessed using a three‐item scale also originally developed in Korean by Chang et al. (2023). A sample item is, “Colleagues make efforts to reduce, reuse, or recycle.” In that study, the scale demonstrated construct validity and showed a Cronbach's alpha of 0.83 (Chang et al. 2023). The alpha value was 0.87 in the current study.

#### 3.2.4 Awareness of Consequences

Awareness of consequences was measured using a seven‐item scale developed by Dalvi‐Esfahani et al. (2017). The research team translated the original scale into Korean and tested its content validity with five experts, comprising two psychology experts (one specializing in psychometrics and one in organizational psychology), two nursing professors, and one doctoral‐prepared clinical nurse, all with experience in translating and adapting research instruments. The Kappa score yielded 1.00, indicating the items’ excellent representation of the construct of awareness of consequences (Polit et al. 2007). We also conducted EFA, which supported a one‐factor structure. An example item is, “Environmental quality will improve if we manage waste more efficiently.” The original scale demonstrated a Cronbach's alpha of 0.76 (Dalvi‐Esfahani et al. 2017); in the present study, the value was 0.88.

#### 3.2.5 Control Variables

Three individual characteristics, age (Katz et al. 2022), conscientiousness (Katz et al. 2022), and direct care provision (de Reeder 2022), were included as control variables, based on prior research that identified their association with pro‐environmental behavior. In the current study, age was self‐reported in years. Conscientiousness was measured using the Korean version of the Big Five Inventory (Kim et al. 2011), originally developed by John and Srivastava (1999). Response options ranged from 1 (*strongly disagree*) to 5 (*strongly agree*), with higher scores indicating greater conscientiousness. In the study by Kim et al. (2011), the Korean scale demonstrated construct and criterion validity, with a Cronbach's alpha of 0.82; in the present study, the value was 0.70. Direct patient care provision was treated as a binary variable (0 = *not providing direct care*, 1 = *providing direct care*). Tenure was not included as a control variable because it was highly correlated with age, raising concerns about multicollinearity; unit type was examined in supplementary analyses but did not substantively alter the results and was, therefore, not retained in the final models.

### 3.3 Data Analysis

We calculated descriptive statistics to summarize the sample characteristics and study variables. To evaluate the assumption of normality, skewness and kurtosis were examined against the recommended thresholds of ±2 and ±7, respectively (Kline 2023). Pearson's and Spearman's correlations were used to examine bivariate relationships. Multicollinearity was evaluated using variance inflation factor (VIF) values, with values greater than 10 indicating potential concern (Hair et al. 2019). We conducted multiple linear regression analysis to examine the associations between green organizational climate, green team climate, awareness of consequences, and green behavior, while controlling for age, conscientiousness, and direct care provision. All statistical analyses were performed using IBM SPSS Statistics for Windows, version 28.0 (IBM Corp., Armonk, NY, USA), with statistical significance set at *p* < 0.05.

### 3.4 Ethical Considerations

Before any data were collected, we explained the study's objectives, procedures, and the voluntary basis of participants’ involvement. Informed consent was obtained electronically from all participants. All data were collected anonymously and stored securely to maintain confidentiality and protect participants’ rights. The Institutional Review Board of Yonsei University Health System approved this study (Approval No: 4‐2025‐0598).

## 4 Results

### 4.1 Sample Characteristics

As shown in Table 1, the sample was predominantly female (95.7%), with a mean age of 33.66 years (*SD* = 7.49). Nurses holding a bachelor's degree or higher constituted 95.5% of the respondents. Participants had an average tenure of 9.64 (*SD* =  7.48) years at the hospital and 5.39 (*SD* =  5.31) years within their unit. Most worked in medical/surgical units (48.5%) or specialty units (31.9%) and provided direct nursing care (91.1%).

**TABLE 1:** Participants’ characteristics (*N* = 649).

Variables	Mean (*SD*)	*n* (%)
Sex		
Male		28 (4.3)
Female		621 (95.7)
Age (years)	33.66 (7.49)	
Education level		
Diploma		29 (4.5)
Bachelor's degree or higher		620 (95.5)
Hospital tenure (years)	9.64 (7.48)	
Unit tenure (years)	5.39 (5.31)	
Unit type		
Medical, surgical, medical–surgical		315 (48.5)
Specialty^a^		207 (31.9)
Others^b^		127 (19.6)
Direct care provision		
Yes		591 (91.1)
No		58 (8.9)

^a^ Specialty units included critical care, emergency, and perioperative units.

^b^ Others included outpatient care units and administrative departments.

Abbreviation: SD, standard deviation.

### 4.2 Descriptive Statistics and Correlations of Study Variables

Table 2 presents the descriptive statistics and the results of bivariate correlation analyses for the study variables. Nurses’ green behavior was positively associated with awareness of consequences (*r* = 0.218, *p* < 0.001), green team climate *(r* = 0.357, *p* < 0.001), and green organizational climate (*r* = 0.256, *p* < 0.001). Among the control variables, age (*r* = 0.205, *p* < 0.001) and conscientiousness (*r* = 0.161, *p* < 0.001) also showed positive correlations with green behavior. Direct care provision was negatively associated with green behavior (*ρ* = –0.145, *p* < 0.001), indicating that nurses involved in direct care reported lower levels of green behavior compared to those not providing direct care.

**TABLE 2:** Means, standard deviations, and correlations of study variables (*N* = 649).

Variable	Mean (*SD*)	*n* (%)	1	2	3	4	5	6
1. Green behavior	3.54 (0.62)		—					
2. Green organizational climate	2.91 (0.85)		0.256^***^	—				
3. Green team climate	2.93 (0.82)		0.357^***^	0.521^***^	—			
4. Awareness of consequences	4.29 (0.48)		0.218^***^	0.008	0.024	—		
5. Age (years)	33.66 (7.49)		0.205^***^	0.078^*^	0.105^**^	0.124^**^	—	
6. Conscientiousness	3.78 (0.56)		0.161^***^	0.108^**^	0.097^*^	0.171^***^	0.168^***^	—
7. Direct care provision^a^		591 (91.1)^b^	−0.145^***^	−0.053	−0.133^***^	−0.030	−0.143^***^	−0.068

*Notes*: Spearman's *ρ* was used to test the correlation between direct care provision and the other study variables. Otherwise, Pearson's *r* was used.

^a^ Direct care provision coded as 0 = not providing direct care, 1 = providing direct care.

^b^ The number and percentage of participants reflect providing direct care as part of their work.

Abbreviation: SD, standard deviation.

^*^ *p* < 0.05, ^**^ *p* < 0.01, ^***^ *p* < 0.001.

### 4.3 Multiple Linear Regression Analysis

Preliminary diagnostics indicated that the assumptions of multiple linear regression were adequately met. Skewness (range: 0.02–0.82) and kurtosis (range: 0.03–0.44) values fell within acceptable thresholds (Kline 2023), and VIFs ranged from 1.03 to 1.40, indicating no concerns regarding multicollinearity (Hair et al. 2019). Multiple linear regression analysis was then conducted to examine how green organizational climate, green team climate, and awareness of consequences relate to nurses’ engagement in green behavior, after accounting for age, conscientiousness, and direct care provision (see Table 3). Green team climate was the strongest predictor (β = 0.274, *p* < 0.001) of green behavior, followed by awareness of consequences (β = 0.181, *p* < 0.001) and green organizational climate (β = 0.090, *p* = 0.030).

**TABLE 3:** Results of multiple linear regression predicting nurses’ green behavior (*N* = 649).

Predictor	B	SE	β	*p*
Age	0.010	0.003	0.126	0.001
Conscientiousness	0.074	0.040	0.067	0.066
Direct care provision	−0.187	0.077	−0.086	0.016
Awareness of consequences	0.235	0.046	0.181	<0.001
Green team climate	0.207	0.031	0.274	<0.001
Green organizational climate	0.065	0.030	0.090	0.030

*Notes*: Direct care provision was coded as 0 = not providing direct care, 1 = providing direct care.

Abbreviations: B, unstandardized regression coefficient; SE, standard error; β, standardized regression coefficient.

## 5 Discussion

### 5.1 Key Findings

This study investigated how nurses’ perceptions of green organizational climate, green team climate, and awareness of consequences were related to their engagement in green behavior. Results indicated that green team climate was the strongest predictor, followed by individual awareness of consequences and green organizational climate. Overall, the findings indicate that nurses’ green behavior is associated with organizational and team climates as well as individual cognitive factors.

It is noteworthy that green team climate was the strongest predictor of nurses’ green behavior. These findings suggest that team‐level social norms, as reflected in co‐workers’ pro‐environmental practices, are important correlates of nurses’ green behavior. Our result aligns with a recent review study linking green team climate to higher eco‐friendly behavior among employees across various industrial settings (Zacher et al. 2023). When co‐workers’ pro‐environmental practices are perceived as normative within the team, employees tend to align their behaviors with those of peers (Norton et al. 2014). This dynamic is particularly relevant in hospitals where collaborative work and informal learning are integral to daily practice (Burm et al. 2019). In these contexts, co‐workers’ visible pro‐environmental actions may function as salient cues that signal and sustain team‐level norms.

Awareness of consequences was the second strongest predictor of green behavior, suggesting that nurses who are more attuned to the negative environmental impacts of their actions are more inclined to engage in green behavior. This finding lends support to the assumptions of the Norm Activation Model (Han 2014; Schwartz 1977), which proposes awareness of consequences as a distal antecedent of pro‐environment behavior. Specifically, when individuals recognize the harmful consequences of their behavior, their sense of responsibility can activate personal norms, which are then associated with a greater likelihood of pro‐environmental actions. Although much of the existing evidence comes from nonclinical contexts (Fu et al. 2020; Li et al. 2023), the present study adds hospital‐based evidence that awareness of consequences is positively associated with nurses’ green behavior.

Consistent with the findings of Aly et al. (2025) and Hasan et al. (2024), perceived green organizational climate was also positively associated with employee green behavior. When employees view environmental sustainability as an organizational priority, green behavior is more likely to be seen as endorsed and professionally supported. However, the association was smaller than that for green team climate or awareness of consequences. One possibility is that organization‐level signals are broad and indirect (Kerrissey et al. 2024); compared with the more tangible cues embedded in team climate, organization‐wide messages can appear abstract and less connected to daily clinical routines (Composto 2025). Although smaller than those for team climate and awareness, the standardized regression coefficient for perceived green organizational climate remained positively associated with employee green behavior, underscoring its continued relevance in clinical settings.

From a practical perspective, although the observed associations were modest in size, they suggest that everyday social and cognitive conditions within nursing work environments may meaningfully relate to nurses’ engagement in green behavior. While direct care provision was included as a control variable, its negative association with green behavior may reflect greater workload and time pressure among nurses providing patient care, leaving fewer resources to attend to environmentally responsible practices. This observation suggests that sustainability initiatives in hospitals may benefit from workload‐sensitive approaches that integrate green practices into routine clinical workflows (Zoromba and El‐Gazar 2025).

### 5.2 Limitations

Several limitations warrant consideration when interpreting the findings of this study. First, the cross‐sectional design limits the ability to infer causality. Second, reliance on self‐reported surveys raises the possibility of social desirability and common method biases; incorporating observational or multisource data would strengthen validity (Hammerton and Munafò 2021). In addition, the open survey distribution strategy prevented the calculation of a response rate and may have introduced selection bias, as nurses with a greater interest in environmental sustainability may have been more likely to participate. Third, the convenience sample drawn from hospitals in South Korea may limit the generalizability of the results to other hospitals and healthcare contexts. Also, although the reliability of the green behavior scale was within acceptable ranges, some items may capture behaviors that depend on organizational infrastructure rather than solely on employees’ voluntary actions. For example, behaviors such as sorting recyclable materials presuppose the availability of appropriate facilities. Consequently, observed scores may partially reflect contextual constraints, which should be considered when interpreting the magnitude of the reported associations. Finally, the predictor set was limited to perceived organizational and team climates, and awareness of consequences; other potentially relevant factors, such as leadership styles (Aly et al. 2025), were not examined. Future research may incorporate leadership behaviors, objective measures of green behavior, longitudinal or multilevel designs, and more diverse samples.

## 6 Conclusion

This study examined the associations between nurses’ perceptions of green organizational climate, green team climate, and awareness of consequences, and their engagement in green behavior. By investigating green behavior in relation to individual awareness and nurses’ perceptions of team and organizational environmental climates, this study extends prior healthcare research, which has tended to emphasize individual‐level determinants. Among the identified factors, green team climate showed a relatively stronger association with green behavior, indicating the salience of co‐worker norms, while awareness of consequences and organizational climate were also positively related to green behavior. These findings underscore the added value of considering social and organizational contexts alongside individual awareness when designing sustainability initiatives in nursing settings. Future research is needed to explore whether the observed associations hold across diverse healthcare settings, organizational contexts, and nursing roles.

## 7 Implications for Nursing Practice and Policy

Our findings suggest several implications for nursing practice and hospital policy. Consistent with the strong association observed between green team climate and nurses’ green behavior, unit‐level environmental initiatives can strengthen shared norms among the team, particularly when they are peer‐led by sustainability champions (Trent et al. 2023). In line with the positive association between awareness of consequences and green behavior, education through short modules and visual reminders on the unit can heighten awareness of the environmental impact of daily tasks and activate individual engagement (Atta et al. 2025; Yang et al. 2024), and brief “green” discussions embedded in routine team huddles can maintain attention and spread tacit know‐how (Agency for Healthcare Research and Quality 2017). Because a green organizational climate was also positively associated with green behavior, hospital‐level policy should institutionalize conditions that support unit‐level norms. Clear organizational signals—embedding sustainability in formal policies and offering time, materials, and recognition—can help sustain behavior change (Health Research & Educational Trust 2014). At the system level, accrediting bodies and payers can integrate hospital‐level environmental metrics into certification, public reporting, and incentives, and offer targeted funding to offset the upfront costs of validated reusables.

## Author Contributions

*Study design*: Seung Eun Lee, Heejin Lim, and Sujin Nam. *Data collection*: Seung Eun Lee and Heejin Lim. *Data analysis*: Seung Eun Lee, Heejin Lim, and Sujin Nam. *Study supervision*: Seung Eun Lee. *Manuscript writing*: Seung Eun Lee, Heejin Lim, and Sujin Nam. *Critical revisions for important intellectual content*: Seung Eun Lee and V. Susan Dahinten.

## Funding

This work was supported by the National Research Foundation of Korea (NRF) grants funded by the Korean government (MSIT) (RS‐2023‐00208138). The funding body has no involvement in study design; collection, management, analysis, and interpretation of data; or the decision to submit for publication.

## Conflicts of Interest

The authors declare that there are no conflicts of interest.

## Data Availability

The data that support the findings of this study are available on request from the corresponding author. The data are not publicly available due to privacy or ethical restrictions.
